# Methylation Analysis of Several Tumour Suppressor Genes Shows a Low Frequency of Methylation of *CDKN2A* and *RARB* in Uveal Melanomas

**DOI:** 10.1002/cfg.295

**Published:** 2003-06

**Authors:** Michael Zeschnigk, Frank Tschentscher, Christina Lich, Birgit Brandt, Bernhard Horsthemke, Dietmar R. Lohmann

**Affiliations:** Institut für Humangenetik Universitätsklinikum Essen Hufelandstrasse 55 Essen 45122 Germany

## Abstract

We have investigated the frequency of methylation of several tumour suppressor genes in uveal melanoma. As the loss of one copy of chromosome 3 (monosomy 3), which is found in about half of these tumours, is tightly associated with metastatic disease, a special emphasis was laid on genes located on this chromosome, including the fragile histidine triad (*FHIT*), von Hippel–Lindau (*VHL*), β-catenin (*CTNNB1*), activated leukocyte cell adhesion molecule (*ALCAM*) and retinoic acid receptor-β2 (*RARB*) genes. In addition, the methylation patterns of the CpG-rich regions 5′ of the E-cadherin (*CDH1*), p16/cyclin-dependent kinase inhibitor 2 A (*CDKN2A*) and retinoblastoma (*RB1*) genes were analysed by bisulphite genomic sequencing or methylation-specific PCR (MSP). Furthermore, the SNRPN and *D15S63* loci, which are located in the imprinted region of chromosome 15, were included in the study. Aberrant methylation was detected in nine of 40 tumours analysed: The imprinted *SNRPN* and *D15S63* loci were hypermethylated in three tumours, all of which retained both copies of chromosome 3. Methylated *RARB* alleles were detected in three tumours, whereas in three other tumours *CDKN2A* was found to be methylated. As we did not find RARB and CDKN2A preferentially methylated in tumours with monosomy 3, which is a significant predictor of metastatic disease, we suggest that these genes may play a causative role in the formation of uveal melanoma but not in the development of metastases.


**Comparative and Functional Genomics**
*Comp Funct Genom* 2003; **4**: 329–336.
Published online in Wiley InterScience (www.interscience.wiley.com). **DOI:** 10.1002/cfg.295

**Primary Research Paper**

### Methylation analysis of several tumour suppressor genes shows a low frequency of methylation of *CDKN2A* and *RARB* in uveal melanomas

Michael Zeschnigk*, Frank Tschentscher, Christina Lich, Birgit Brandt, Bernhard Horsthemke and Dietmar R. Lohmann
*Institut für Humangenetik, Hufelandstrasse 55, 45122 Essen, Germany*

**Correspondence to:
Michael Zeschnigk, Institut für Humangenetik, Universitätsklinikum Essen, Hufelandstrasse 55, 45122 Essen, Germany.
E-mail: Michael.Zeschnigk@uni-essen.de*

Accepted: 1 April 2003

### Abstract

**We have investigated the frequency of methylation of several tumour suppressor genes in uveal melanoma. As the loss of one copy of chromosome 3 (monosomy 3), which is found in about half of these tumours, is tightly associated with metastatic disease, a special emphasis was laid on genes located on this chromosome, including the fragile histidine triad (*FHIT*), von Hippel–Lindau (*VHL*), $\beta$-catenin (*CTNNB1*), activated leukocyte cell adhesion molecule (*ALCAM*) and retinoic acid receptor-$\beta 2$ (*RARB*) genes. In addition, the methylation patterns of the CpG-rich regions $5'$ of the E-cadherin (*CDH1*), p16/cyclin-dependent kinase inhibitor 2 A (*CDKN2A*) and retinoblastoma (*RB1*) genes were analysed by bisulphite genomic sequencing or methylation-specific PCR (MSP). Furthermore, the *SNRPN* and *D15S63* loci, which are located in the imprinted region of chromosome 15, were included in the study. Aberrant methylation was detected in nine of 40 tumours analysed: The imprinted *SNRPN* and *D15S63* loci were hypermethylated in three tumours, all of which retained both copies of chromosome 3. Methylated *RARB* alleles were detected in three tumours, whereas in three other tumours *CDKN2A* was found to be methylated. As we did not find *RARB* and *CDKN2A* preferentially methylated in tumours with monosomy 3, which is a significant predictor of metastatic disease, we suggest that these genes may play a causative role in the formation of uveal melanoma but not in the development of metastases. Copyright © 2003 John Wiley & Sons, Ltd.**

**Keywords:** uveal melanoma; metastatic disease; tumour suppressor gene; DNA methylation; chromosome 3

### Introduction

Uveal melanoma is the most common form of primary eye cancer, with an incidence of six cases per million people per year. About 50% of these tumours show loss of an entire chromosome 3. This chromosomal aberration is significantly associated with metastatic disease. In long-term studies, approximately 70% of patients with monosomy 3 in the primary tumour died of metastases, whereas tumours having two normal copies of chromosome 3 (disomy 3) rarely gave rise to metastatic disease (Prescher *et al.*, 1998; White *et al.*, 1998a; Sisley *et al.*, 1997). The molecular mechanisms underlying uveal melanoma development, progression and metastasis are yet unknown. Assuming that loss of one chromosome 3 is part of a two-step inactivation mechanism typical of tumour suppressor genes (TSGs), one would expect that metastasizing uveal melanoma depends on biallelic inactivation of one or more genes located on chromosome 3. This hypothesis is backed by the finding of common regions of deletion overlap in tumours that have

Copyright © 2003 John Wiley & Sons, Ltd.

lost only parts of chromosome 3 (Tschentscher *et al.*, 2001). Alternatively, as uveal melanomas occasionally show isodisomy (two identical copies) of chromosome 3, the possible role of epigenetic mechanisms has been put forward (White *et al.*, 1998b). However, no imprinted regions have been reported on chromosome 3 so far.

*De novo* DNA methylation is an epigenetic alteration that has been found to contribute to the development and progression of several tumours (for review, see Baylin *et al.*, 2001). Aberrant methylation frequently targets CpG-rich regions (CpG islands) at the 5′ end of genes. With the exception of CpG islands associated with imprinted genes, or genes located on the inactive X chromosome, these regions are maintained free of methylation in normal cells. The *RB1* gene, which was the first TSG shown to be methylated in tumours (Greger *et al.*, 1989; Sakai *et al.*, 1991), shows allele-specific epigenetic alterations in about 10% of retinoblastomas (Klutz *et al.*, 1999). Promoter methylation can result in the inactivation of genes, and therefore putative TSGs can be identified using methylation analysis of tumour DNA.

To analyse the role of DNA methylation in uveal melanoma, we have analysed the methylation status of the known or putative promoter/exon 1 regions of eight genes known to be involved in tumourigenesis, and two imprinted loci, in 40 uveal melanomas. In view of the strong association of monosomy 3 with metastatic disease, the methylation status of genes on this chromosome was of particular interest. We investigated the fragile histidine triad gene (*FHIT*), a putative TSG located in chromosome region 3p14.2, the retinoic acid receptor-$\beta$2 gene (*RARB*, on 3p24), the *VHL* gene (3p25), the activated leukocyte cell adhesion molecule gene (*ALCAM*, on 3q13) and the $\beta$-catenin gene (*CTNNB1*, on 3p21). We also included the E-cadherin gene (*CDH1*, on 16q22) in our study, as loss of function of the complex formed by $\beta$-catenin and E-cadherin occurs in a variety of epithelial tumours and is correlated with invasion and metastasis (Yap, 1998). In addition, *CDH1* was found to be expressed in a variable manner in uveal melanoma (Anastassiou *et al.*, 2001).

We also analysed the methylation status of the *RB1* (13q14) and *CDKN2A* (9p21) genes, which are involved in tumourigenesis of several tumours, including uveal melanoma. In previous studies, *CDKN2A* methylation was reported in 6% (Merbs and Sidransky, 1999) to 34% (Van der Velden *et al.*, 2001) of uveal melanomas. Recently, loss of heterozygosity (LOH) at the *RB1* locus was found to be a common alteration (21%) in uveal melanomas (Scholes *et al.*, 2001), suggesting a role for *RB1*, or additional loci close to *RB1*, in uveal melanoma tumourigenesis.

Finally, we investigated the *SNRPN* and *D15S63* loci, which are located in the imprinted region of chromosome 15. This region, which is affected in patients with Prader–Willi or Angelman syndromes, is not known to play a role in tumour formation but we were interested to know whether imprint maintenance is affected in tumour cells.

### Materials and methods

### Patients and biopsy specimens

Diagnosis of uveal melanoma was established following current ophthalmological and histological criteria. Vital tumour samples were obtained from patients treated by primary enucleation without prior radiation or chemotherapy. Peripheral blood and tumour material were obtained at the time of operation and stored at −80 °C.

### Analysis of chromosome 3 status

DNA extractions from blood and tumour samples and PCR-based diagnosis of chromosome 3 loss were carried out as described previously (Tschentscher *et al.*, 2000; 2001). In brief, fluorescently labelled primers were used for amplification of the microsatellites *D3S3050, D3S2406, D3S3045, D3S1744, D3S2421, D3S1311* and *D3S1272* from the DNA of tumours and corresponding blood samples, in individual reactions. Reaction products were analysed using an ABI 3100 automated capillary genetic analyser and the GeneScan™ and Genotyper® software (Applied Biosystems, Foster City, CA). According to the report by Tschentscher *et al.* (2000), uveal melanomas showing LOH for all informative markers on chromosome 3 were considered to have monosomy 3.

### Bisulphite treatment

Bisulphite modification was performed as described (Clark *et al.*, 1994). Briefly, tumour DNA (5 µg

Copyright © 2003 John Wiley & Sons, Ltd.

in 50 μl) was denatured by adding 6 μl freshly prepared 3 M NaOH and incubating the solution at 37 °C for 15 min. For complete denaturation, the samples were incubated at 95 °C for 1 min and subsequently cooled on ice. The bisulphite solution was prepared by dissolving 8.1 g sodium bisulphite in 16 ml $H_2O$, adding 1 ml 40 mM hydrochinone solution and adjusting the pH to 5.0 with 600 μl 10 M NaOH; 0.5 ml bisulphite solution was mixed with the denatured DNA, overlaid with mineral oil, and incubated at 55 °C for 16–18 h in a water bath in the dark. The DNA was recovered using the Wizard DNA Clean-Up System (Promega, Mannheim, Germany) and eluted in 100 μl $H_2O$. Following this, 11 μl 3 M NaOH was added and the sample was incubated for 15 min at 37 °C. The solution was then neutralized by adding 110 μl 6 M $NH_4OAc$, pH 7.0. The DNA was ethanol-precipitated, washed in 70% ethanol, dried and resuspended in 40 μl $H_2O$. Bisulphite treated DNA (2 μl) was used in each of the PCR assays.

### Methylation-specific PCR (MSP)

Established MSP protocols were used to analyse the methylation status of the *VHL, CDH1, CDKN2A* (primers p16-U2 and p16-M2; for sequence, see Herman *et al*., 1996) and *RB1* promoter/exon 1 regions, as well as the imprinted *SNRPN* and *D15S63* loci (Herman *et al*., 1996; Zeschnigk *et al*., 1997, 1999; Runte *et al*., 2001). The *RARB*-MSP was modified from Widschwendter *et al.* (2000). PCR was performed in a 25 μl reaction volume containing 2 μl bisulphite-treated DNA. The conditions were 10 mM Tris–HCl, pH 8.3, 50 mM KCl, 1.5 mM $MgCl_2$, 50 μM of each dNTP and 0.5 U AmpliTaq (Applied Biosystems, Foster City, USA). For the detection of methylated alleles, 1 μM each primer (RAR–MAS and RAR–MES) was used; for the detection of unmethylated alleles, we used 1 μM each primer (RAR–UAS and RAR–USE) in a separate reaction. After the initial denaturation step at 94 °C for 5 min, 35 cycles of denaturation (at 95 °C for 15 s), annealing (at 60 °C for 30 s) and extension (at 72 °C for 30 s) were performed, followed by a final extension (at 72 °C for 5 min). MSP products were separated on 2.5% agarose gels and visualized by ethidium bromide staining. The primer sequences used were as follows: RAR–MSE: 5′-ATGTCGAGAACGCGAGCGATTC-3′ (nt 102–123); RAR–MAS: 5′-CTCGACCAATCCAACCGAAACG-3′ (nt 232–253); RAR–USE: 5′-GGATGTTGAGAATGTGAGTGATTT-3′ (nt 100–123); RAR–UAS: 5′-TACTCAACCAATCCAACCAAAACA-3′ (nt 232–255). The nucleotide positions of the primers are given according to Widschwendter *et al.* (2000).

### Genomic sequencing

PCRs for the *ALCAM* analysis were performed with the primers ALCAM5′ and ALCAM3′ (Table 1), which were designed to bind to the putative promoter/exon 1 region (GenBank Accession Nos L38608 and AC078806) in bisulphite-treated DNA. The primer sequences for *CTNNB1* (CTNNB5′ and CTNNB3′) and *FHIT* (FHITas5′ and FHITas3′) amplification were generated on the basis of published sequence data (GenBank Accession Nos X89448 and U76262/U76263, respectively). These primer sequences are all given in Table 1. PCRs for methylation analysis of these genes were performed under the conditions given above for the

**Table 1.** The primers used for genomic sequencing

| Primer | Sequence | Accession | Position (nt) | Annealing (C°) |
|---|---|---|---|---|
| FHITas5′ | GTTTTTATTTTTTAGGATGTTGATAGTTGG | U76262 | 1413–11442 | 60 |
| FHITas3′ | AATTACTATCACTATAACTTTCAATTAACC | U76263 | 129–158 | 60 |
| CTNNB5′ | TATTTTAAGTTTTTc/tGGTTTGTGGTAGTAG | X89448 | 990–1020 | 64 |
| CTNNB3′ | CAAACACCTCAAAAAAACAAACTCCTCC | X89448 | 1142–1170 | 64 |
| ALCAM5′ | ATTATTTAAGTGTTTTTTGGAAATAGAGGG | AC078806 | 73938–73968 | 64 |
| ALCAM3′ | TTCCATATTCCTCCTCCTTCTTAATAAACC | AC078806 | 74217–74247 | 64 |

All primer sequences are given in the 5′ to 3′ orientation. The primers were designed based on the sequence information (accession) provided by GenBank database at NCBI. The primer positions (position) according to the GenBank data file and annealing temperatures (annealing) used for PCR are also given. c/t, C and T were added in equal concentration during primer synthesis.

Copyright © 2003 John Wiley & Sons, Ltd. 

RARB–MSP; however, the primer concentrations were adjusted to 0.8 μM each. After the initial denaturation step (at 94 °C for 5 min), 35 cycles of denaturation (at 95 °C for 15 s), annealing (at 60 °C for the *FHIT* PCR and 64 °C for the *ALCAM* and *CTNNB* PCR for 15 s) and extension (at 72 °C for 15 s) were performed, followed by a final extension step (at 72 °C for 5 min). PCR products were separated on 2.5% agarose gels and visualized by ethidium bromide staining. Agarose gel slices containing PCR products were excised from the gels and purified using the MiniElute Extraction Kit (Qiagen, Hilden, Germany).

### DNA sequencing

*ALCAM, FHIT* and *CTNNB* PCR products were sequenced using an ABI 3100 automated capillary genetic analyser (Applied Biosystems, Foster City, CA), using the corresponding 3′ primers as sequencing primers. To enable the SequenceAnalyse™ software for automated analysis, 1 μl Matrix Standard Set DS-01 (Applied Biosystems, Foster City, CA) was added to each sample before loading it onto the machine. As the sequence reactions were started with the 3′ primer, formerly methylated cytosines were indicated by a G-peak in the electropherogram.

### Results

Established MSP protocols were used to analyse the methylation status of the *VHL, CDH1, CDKN2A, RARB,* and *RB1* promoter/exon 1 regions as well as the imprinted *SNRPN* and *D15S63* loci (Herman *et al.*, 1996; Zeschnigk *et al.*, 1997, 1999; Runte *et al.*, 2001). The accuracy of MSP was controlled by the use of methylated control DNA as a template. To determine the methylation patterns of the *FHIT, CTNNB1* and *ALCAM* genes, we generated PCR primers (Table 1) that hybridize to bisulphite-treated DNA in the corresponding promoter/exon 1 regions. After performing PCR on bisulphite-modified genomic tumour DNA and methylated control templates, the PCR products were purified and sequenced. The use of premethylated DNA as a control confirmed that all of the assays were capable of detecting methylated alleles.

The methylation status of all 10 loci was tested in 40 tumour samples, 50% of which had monosomy 3. Detection of any methylation in non-imprinted CpG islands is described as methylated. As summarized in Table 2, none of the tumours showed methylation in the promoter/exon 1 regions of the *FHIT, CTNNB1, VHL, ALCAM, CDH1* and *RB1* genes. Two tumours with disomy 3 (T21 and T22) and one tumour with monosomy 3 (T15) showed methylation of the *RARB* gene on chromosome 3 (Figure 1a). In addition to a methylated allele, the MSP displayed the presence of an unmethylated allele in these tumours. The observation of two *RARB* alleles appears to be in conflict with the presence of monosomy 3 in tumour T15, but could be explained by methylation mosaicism.

The *CDKN2A* promoter region was methylated in three different tumours, one tumour with monosomy 3 (T16) and two tumours with disomy 3 (T25, T36) (Figure 1b). In all three tumours, in addition to the methylated allele, a PCR product specific for an unmethylated allele was present.

The imprinted loci *SNRPN* and *D15S63* are known to be methylated on the maternal chromosome, and unmethylated on the paternal chromosome, in normal individuals. Surprisingly, the PCR product specific for the unmethylated *D15S63* alleles (143 bp) was absent in two tumours (T27 and T35), and a third tumour (T24) showed only a weak signal. In one of these tumours (T27), the PCR product specific for the unmethylated *SNRPN* allele (221 bp) was also reduced (Figure 2). These results are compatible with the assumption of *de novo* methylation of the paternal *D15S63* alleles in the tumours. Alternatively, they may be explained by loss of the paternal *D15S63* allele. However, LOH is unlikely for tumours T24 and T35, because both show two differentially methylated alleles at the closely linked *SNRPN* locus. It is noteworthy that the altered methylation of the *SNRPN/D15S63* region was only observed in tumours with disomy 3.

In summary, aberrant methylation was detected in nine out of 40 primary uveal melanomas and was restricted to the TSGs *RARB* and *CDKN2A* and the imprinted region on chromosome 15. Altered methylation was less frequent in tumours with monosomy 3 (2/20) than in tumours with disomy 3 (7/20).

Copyright © 2003 John Wiley & Sons, Ltd. 

**Table 2.** The methylation status of 10 genes/loci in 40 uveal melanomas

| Genes/loci | Uveal melanomas with monosomy 3 | | | | | | | | | | | | | | | | | | |
|---|---|---|---|---|---|---|---|---|---|---|---|---|---|---|---|---|---|---|---|
| | T1 | T2 | T3 | T4 | T5 | T6 | T7 | T8 | T9 | T10 | T11 | T12 | T13 | T14 | T15 | T16 | T17 | T18 | T19 |
| *FHIT* | − | − | − | − | − | − | − | − | − | − | − | − | − | − | − | − | − | − | − |
| *CTNNB* | − | − | − | − | − | − | − | − | − | − | − | − | − | − | − | − | − | − | − |
| *VHL* | − | − | − | − | − | − | − | − | − | − | − | − | − | − | − | − | − | − | − |
| *ALCAM* | − | nd | − | − | − | − | − | − | − | − | − | − | − | − | − | − | − | − | nd |
| *CDH1* | − | − | − | − | − | − | − | − | − | − | − | − | − | − | − | − | − | − | − |
| *RB1* | − | − | − | − | − | − | − | − | − | − | − | − | − | − | − | − | − | − | − |
| *RARB* | − | − | − | − | − | − | − | − | − | − | − | − | − | − | + | − | − | − | − |
| *CDKN2A* | − | − | − | − | − | − | − | − | − | − | − | − | − | − | − | + | − | − | − |
| *SNRPN* | n | n | n | n | n | n | n | n | n | n | n | n | n | n | n | n | n | n | n |
| *D15S63* | n | n | n | n | n | n | n | n | n | n | n | n | n | n | n | n | n | n | n |

| Genes/loci | Uveal melanomas with disomy 3 | | | | | | | | | | | | | | | | | | | | |
|---|---|---|---|---|---|---|---|---|---|---|---|---|---|---|---|---|---|---|---|---|---|
| | T20 | T21 | T22 | T23 | T24 | T25 | T26 | T27 | T28 | T29 | T30 | T31 | T32 | T33 | T34 | T35 | T36 | T37 | T38 | T39 | T40 |
| *FHIT* | − | − | − | − | − | − | − | − | − | − | − | − | − | − | − | − | − | − | nd | − | − |
| *CTNNB* | − | − | − | − | − | − | − | − | − | − | − | − | − | − | − | nd | − | − | − | − | − |
| *VHL* | − | − | − | − | − | − | − | − | − | − | − | − | − | − | − | − | − | − | − | − | − |
| *ALCAM* | nd | − | − | − | − | − | − | − | − | − | − | − | − | − | − | − | − | − | − | − | − |
| *CDH1* | − | − | − | − | − | − | − | − | − | − | − | − | − | − | − | − | − | − | − | − | − |
| *RB1* | − | − | nd | − | − | − | − | − | − | − | − | − | − | − | − | − | − | − | − | − | − |
| *RARB* | − | + | + | − | − | − | − | − | − | − | − | − | − | − | − | − | − | − | − | − | − |
| *CDKN2A* | − | − | − | − | − | + | − | − | − | − | − | − | − | − | − | − | + | − | − | − | − |
| *SNRPN* | n | n | n | n | n | n | n | + | n | n | n | n | n | n | n | n | n | n | n | n | n |
| *D15S63* | n | n | n | n | + | n | n | + | n | n | n | n | n | n | n | + | n | n | n | n | n |

n, normal methylation pattern; +, methylation/loss of unmethylated allele; −, no detectable methylation; nd, not done.

### Discussion

To identify TSGs in uveal melanoma, we analysed the methylation status of the promoter/exon 1 regions of a set of TSGs in 40 tumour samples. As the loss of chromosome 3 in this tumour is associated with metastatic disease and poor survival, we focused our study on genes located on chromosome 3. No methylation was present in the CpG islands of the *VHL, FHIT, CTNNB1* and *ALCAM* genes, which are located on chromosome 3. While methylation-mediated silencing of *CTNNB1* and *ALCAM* have not previously been reported in any tumour, *VHL* and *FHIT* are known to be inactivated by promoter methylation in a wide range of other tumour entities, indicating that this alteration is a common inactivation mechanism of both TSGs. The absence of this epigenetic modification in 40 uveal melanomas makes a major role of *VHL* und *FHIT* in uveal melanoma tumourigenesis unlikely.

Three uveal melanomas showed methylation of *RARB*, which is thought to function as a TSG (Liu *et al.*, 1996; Houle *et al.*, 1993). This gene was shown to be inactivated by methylation in 38% of primary breast cancers Widschwendter *et al.* (2000) and 40% of primary non-small cell lung cancers (Zöchbauer-Müller *et al.*, 2001). Although the frequency of *RARB* methylation is much lower in uveal melanomas (7.5%), our results suggest that *RARB* may be involved in uveal melanoma formation. However, it is unlikely that *RARB* is a major gene involved in metastatic disease in tumours with monosomy 3, because two of the three uveal melanomas with methylation of *RARB* have disomy 3, and methylation in the tumour with monosomy 3 is present in only some of the cells.

Among the 40 uveal melanomas investigated here, three (7.5%) showed methylation of *CDKN2A*. The frequency of *CDKN2A* methylation in our study corresponds well to the findings of Merbs *et al.* (1999), who identified methylated *CDKN2A* alleles in two out of 33 uveal melanomas (6%), but it is in contrast to the results of van der Velden *et al.* (2001), who investigated 22 tumours and found *CDKN2A* methylation in 34% of them. In all three studies, MSP was used to determine the methylation status, and we have no explanation for the discrepancy between these results. Moreover, van der Velden *et al.* found an association between

Copyright © 2003 John Wiley & Sons, Ltd. 

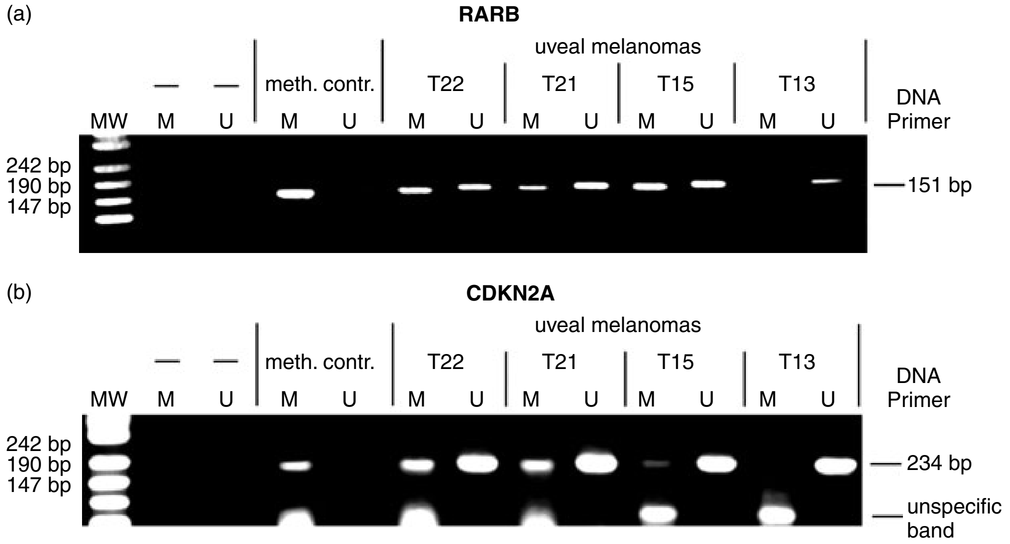

**Figure 1.** Methylation-specific PCR of bisulphite treated primary uveal melanoma DNAs (T) and methylated control DNA (meth. contr.) using primers specific for methylated (M) and unmethylated (U) DNA, respectively. A PCR product in lanes marked with a U indicates the presence of an unmethylated allele, and in lanes marked with an M indicates the presence of a methylated allele. The PCR product sizes of the *RARB* (a) and *CDKN2A* (b) alleles are 152 bp and 234 bp, respectively. Marker (MW) = MspI-digested pUC19 DNA; PCR products were separated on a 2.5% agarose gel

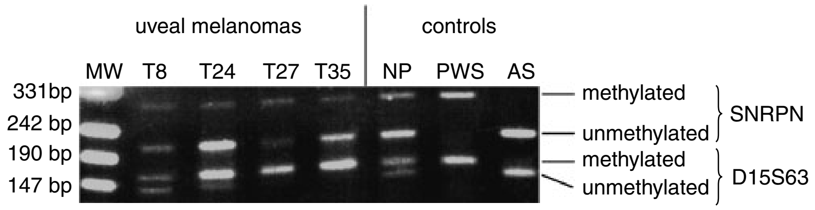

**Figure 2.** Analysis of the imprinted *SNRPN* and *D15S63* loci. A single-tube MSP assay was performed on bisulphite-treated primary uveal melanoma DNAs (T) and DNA from blood cells from Prader–Willi (PWS) or Angelman syndrome (AS) patients and a normal control (NP). A normal methylation status is reflected by two PCR products with sizes 221 bp and 313 bp for the *SNRPN,* and two PCR products of 143 bp and 161 bp for the *D15S63* loci. Marker (MW) = MspI-digested pUC19 DNA. The MSP products were separated on 3% agarose gels

*CDKN2A* methylation and tumour location and presence of epithelioid cells, which are prognostic factors for metastatic disease. In our tumour set, however, *CDKN2A* methylation was not associated with monosomy 3, which is the best predictor of metastatic disease known so far.

Recently, LOH at a polymorphic locus within the *RB1* gene was found in five (21%) out of 27 uveal melanomas, suggesting that this gene is involved in the pathogenesis of uveal melanoma (Scholes *et al.*, 2001). In retinoblastoma, a tumour that invariably shows biallelic inactivation of the *RB1* gene, methylation is observed in 10% of tumours. As we did not find methylated *RB1* alleles in 40 analysed samples, this gene is most likely not the TSG targeted by the LOH at 13q14 in uveal melanoma. As discussed by Scholes *et al.* (2001), a locus closely linked to, but distinct from, *RB1* may be involved.

In several tumour types, the loss of E-cadherin is associated with invasive disease and metastasis. In uveal melanomas, this gene was found to show varying levels of expression, at both the RNA and the protein level (Anastassiou *et al.*, 2001). As we found no methylated *CDH1* alleles among 40 tumours, the low expression of this gene in some uveal melanomas is most probably not caused by promoter methylation.

Copyright © 2003 John Wiley & Sons, Ltd. 

We found alterations in the DNA methylation of the imprinted region on chromosome 15 in three uveal melanomas, all with disomy 3. In one tumour (T27), both *SNRPN* and *D15S63* appeared to be hypermethylated. In principle, the absence of an unmethylated allele in the tumours can be due to loss of heterozygosity; however, the presence of two differentially methylated alleles at the closely linked *SNRPN* locus in tumour T24 and T35 shows that altered *D15S63* methylation is the likely cause of the observed results in these tumours. Interestingly, alterations of the *SNRPN/D15S63* methylation pattern were restricted to tumours with disomy 3, which correlates with a good prognosis. In a previous study (Dittrich *et al*., 1993), we found various degrees of *D15S63* hypomethylation in different tumours. As *D15S63* and *SNRPN* are unlikely to play a role in tumour formation, it is likely that these changes reflect loss of imprint maintenance caused by tumour formation.

In summary, our data indicate that methylation of known tumour suppressor genes is infrequent in uveal melanoma. Specifically, several genes on chromosome 3 are not preferentially methylated in tumours with monosomy 3. However, we found methylation of the TSGs *CDKN2A* and *RARB* in 7.5% of primary tumours. While promoter methylation, as well as homozygous deletions, of *CDKN2A* have been reported previously in uveal melanomas (Merbs and Sidransky, 1999; van der Velden *et al*., 2001), this is the first report showing methylation alterations of the *RARB* gene in this tumour. Therefore, we suggest that this gene may play a role in uveal melanoma formation.

### Acknowledgements

We thank Vera Trappe and Bianca Beyer for excellent technical assistance and Jennifer Berger for providing tumour DNA. This work was supported by the Deutsche Forschungsgemeinschaft (LO530/3-3).

### References

Anastassiou G, Tschentscher F, Zeschnigk M, Bornfeld N. 2001. Cadherin expression in Uveal Melanoma. *Exp Eye Res* (in press).

Baylin SB, Esteller M, Rountree MR, *et al*. 2001. Aberrant patterns of DNA methylation, chromatin formation and gene expression in cancer. *Hum Mol Genet* **10**: 687–692.

Clark SJ, Harrison J, Paul CL, Frommer M. 1994. High sensitivity mapping of methylated cytosines. *Nucleic Acids Res* **22**: 2990–2997.

Dittrich B, Buiting K, Gross S, Horsthemke B. 1993. Characterization of a methylation imprint in the Prader–Willi syndrome chromosome region. *Hum Mol Genet* **2**: 1995–1999.

Greger V, Passarge E, Hopping W, Messmer E, Horsthemke B. 1989. Epigenetic changes may contribute to the formation and spontaneous regression of retinoblastoma. *Hum Genet* **83**: 155–158.

Herman JG, Graff JR, Myohänen S, Nelkin BD, Baylin B. 1996. Methylation-specific PCR: a novel PCR assay for methylation status of CpG islands. *Proc Natl Acad Sci USA* **93**: 9821–9826.

Houle B, Rochette-Egly C, Bradley WE. 1993. Tumour-suppressive effect of the retinoic acid receptor $\beta$ in human epidermoid lung cancer cells. *Proc Natl Acad Sci USA* **90**: 985–989.

Klutz M, Horsthemke B, Lohmann DR. 1999. RB1 gene mutations in peripheral blood DNA of patients with isolated unilateral retinoblastoma. *Am J Hum Genet* **64**: 667–668.

Liu Y, Lee MO, Wang HG, *et al*. 1996. Retinoic acid receptor $\beta$ mediates the growth-inhibitory effect of retinoic acid by promoting apoptosis in human breast cancer cells. *Mol Cell Biol* **16**: 1138–1149.

Merbs SL, Sidransky D. 1999. Analysis of p16 (CDKN2/MTS-1/INK4A) alterations in primary sporadic uveal melanoma. *Invest Ophthalmol Vis Sci* **40**: 779–783.

Prescher G, Bornfeld N, Hirche H, *et al*. 1996. Prognostic implications of monosomy 3 in uveal melanoma. *Lancet* **347**: 1222–1225.

Runte M, Färber C, Lich C, *et al*. 2001. Comprehensive methylation analysis in typical and atypical PWS and AS patients with normal biparental chromosomes 15. *Eur J Hum Genet* **9**: 519–526.

Sakai T, Toguchida J, Ohtani N, *et al*. 1991. Allele-specific hypermethylation of the retinoblastoma tumour-suppressor gene. *Am J Hum Genet* **48**: 880–888.

Scholes AG, Liloglou T, Maloney P, *et al*. 2001. Loss of heterozygosity on chromosomes 3, 9, 13 and 17, including the retinoblastoma locus, in uveal melanoma. *Invest Ophthalmol Vis Sci* **42**: 2472–2477.

Sisley K, Rennie IG, Parsons MA, *et al*. 1997. Abnormalities of chromosomes 3 and 8 in posterior uveal melanoma correlate with prognosis. *Genes Chromosomes Cancer* **19**: 22–28.

Tschentscher F, Prescher G, Horsman DE, *et al*. 2001. Partial deletions of the long and short arm of chromosome 3 point to two tumour suppressor genes in uveal melanoma. *Cancer Res* **61**: 3439–3442.

Tschentscher F, Prescher G, Zeschnigk M, Horsthemke B, Lohmann DR. 2000. Identification of chromosomes 3, 6 and 8 aberrations in uveal melanoma by microsatellite analysis in comparison to comparative genomic hybridization. *Cancer Genet Cytogenet* **122**: 13–17.

van der Velden PA, Metzelaar-Blok JA, Bergman W, *et al*. 2001. Promoter hypermethylation: a common cause of reduced p16(INK4a) expression in uveal melanoma. *Cancer Res* **61**: 5303–5306.

White VA, McNeil BK, Horsman DE. 1998. Acquired homozygosity (isodisomy) of chromosome 3 in uveal melanoma. *Cancer Genet Cytogenet* **102**: 40–45.

White VA, Chambers JD, Courtright PD, Chang WY, Horsman DE. 1998. Correlation of cytogenetic abnormalities with the outcome of patients with uveal melanoma. *Cancer* **83**: 354–359.

Copyright © 2003 John Wiley & Sons, Ltd. 

Widschwendter M, Berger J, Hermann M, *et al*. 2000. Methylation and silencing of the retinoic acid receptor-$\beta$2 gene in breast cancer. *J Natl Cancer Inst* **92**: 826–832.

Yap AS. 1998. The morphogenetic role of cadherin cell adhesion molecules in human cancer. *Cancer Invest* **16**: 252–261.

Zeschnigk M, Lich C, Buiting K, Doerfler W, Horsthemke B. 1997. A single-tube PCR test for the diagnosis of Angelman and Prader–Willi syndrome based on allelic methylation differences at the SNRPN locus. *Eur J Hum Genet* **5**: 94–98.

Zeschnigk M, Lohmann D, Horsthemke B. 1999. A PCR test for the detection of hypermethylated alleles at the retinoblastoma locus. *J Med Genet* **36**: 793–794.

Zöchbauer-Müller S, Fong KM, Virmani AK, *et al*. 2001. Aberrant promoter methylation of multiple genes in non-small cell lung cancers. *Cancer Res* **61**: 249–255.

Copyright © 2003 John Wiley & Sons, Ltd. 
